# Exploring disciplinary perspectives on community resilience

**DOI:** 10.1111/disa.70036

**Published:** 2025-12-22

**Authors:** Alan Forster, Leigh‐Anne Hepburn, Liz Brogden, Megan Boston, Laurent Galbrun, Taibat Lawanson, Jolanda Morkel, Des Bernie

**Affiliations:** ^1^ Heriot‐Watt University United Kingdom; ^2^ The University of Sydney Australia; ^3^ The University of Queensland Australia; ^4^ The University of Waikato New Zealand; ^5^ The University of Liverpool United Kingdom; ^6^ STADIO Higher Education South Africa; ^7^ Icecream Architecture United Kingdom

**Keywords:** built environment, community resilience, design, education, transdisciplinary

## Abstract

Throughout human history, communities have responded to challenges in urban and rural contexts by engaging multiple agents and actors, including individuals, institutions, and governments. Disciplinary expertise, including deep knowledge and practice, has contributed to economic, social, technological, and political change. Yet, it is increasingly apparent that the complex global, systems‐level challenges facing twenty‐first century communities require responses that transcend traditional disciplinary boundaries. The ability of communities to respond to challenges faced, from natural and anthropogenic hazards to the systemic threat of climate change, is often referred to as ‘community resilience’. Despite increasing scholarly interest, there appears to be, however, a lack of consistency in understanding and applying community resilience among cross‐disciplinary practitioners. This ambiguity can limit the potential of collaborative action and impact at the community level. This study explores cross‐disciplinary perspectives of community resilience to better understand how the term is described and applied in practice. Drawing on the experiences of more than 100 international respondents to an online survey, this study analyses the emerging themes to gauge the potential of transdisciplinary community resilience in realising the possible value of collective action.

## 1 INTRODUCTION

This introduction establishes the rationale and objectives for the research. It creates context for the work by outlining five critical aspects of community resilience: (i) understanding resilience: definitions and evolution; (ii) defining community in resilience contexts; (iii) critical perspectives on community resilience; (iv) practice‐based versus academic approaches; and (v) the case for transdisciplinary approaches. These fundamental aspects of community resilience are discussed below (followed by a subsection on the aims and contribution of the study), and supported by current literature in the field.

### 1.1 Understanding resilience: definitions and evolution

Resilience can be described as the ability to respond to complex challenges, underpinned by notions of resistance, robustness, prevention, and recovery from a loss of functionality (Bruneau et al., 2003; Norris et al., 2008). When considered in a built environment context, community resilience can be described as the ability to adapt or transform in the face of disruption (Folke et al., 2002; Magis, 2010; Berkes and Ross, 2013) and ‘a process linking a network of adaptive capacities … to adaptation after a disturbance or adversity’ (Norris et al., 2008, p. 127). These definitions expand on Holling's (1973, p. 14) seminal definition of resilience as ‘the ability of ecological systems to respond to external shocks’. More recently, scholars have focused on the interrelationships between physical and social factors of resilience (Sherrieb, Norris, and Galea, 2010; South et al., 2020; Nofal and van de Lindt, 2022), recognising that sustained and proactive approaches to learning can support communities ‘to adapt to future shocks and vulnerabilities’ (Koliou et al., 2020, p. 132).

Norris et al. (2008) provide a framework that shifts our understanding of community resilience from simply ‘bouncing back’, towards one that harnesses adaptive capacity across four key domains: economic development; social capital; information and communication; and community competence. In this way, resilience is conceptualised as a process rather than an outcome, supporting communities in learning, adapting, and transforming in the face of adversity. This framework is of particular interest to this study given the transdisciplinary approach, acknowledging that community resilience emerges at the intersection of multiple systems, disciplines, and knowledge domains.

### 1.2 Defining community in resilience contexts

It is also important to define the concept of community itself, recognising that the interplay of both social and spatial dimensions reflects how resilience is understood. Berkes and Ross (2013) describe community as a social unit and as a place‐based entity. In the first description, community is defined by its relationships and shared identity, the social networks, norms, and sense of belonging that emerge through connection. In the second description, community is defined by the physical or geographic boundaries that differentiate locations as well as the shared infrastructure, resources, and proximity that materialise in such spaces. This paper adopts an integrative understanding of community that includes the social networks and relationships of residents, and the built environments they inhabit. In this way, we can understand community resilience as emerging from the interaction between the relational and the physical.

### 1.3 Critical perspectives on community resilience

The concept of community resilience is, however, not without significant critique. Scholars have questioned the political implications of resilience discourse, in particular its potential to reinforce existing inequalities rather than address systematic and structural barriers. Kaika (2017) argues that the resilience rhetoric shifts the responsibility for change from institutions to communities, placing undue pressure on communities to respond to, or bounce back from, challenges they did not create. Other scholars such as Vale (2014) surface the unintended or exclusionary impact of resilience politics, asking whose resilience is being prioritised and calling for a more critical and inclusive approach to community resilience that better reflects the needs and desires of community, while also acknowledging the power dynamics at play. Furthermore, as noted by Uekusa and Cretney (2022), prolonged exposure to one or more disasters can create resilience, fatigue, and frustration when only positive attributes are publicly shared, overlooking the daily and continued struggles of individuals within the community.

A review of the extant literature (Boston et al., 2024) suggests a lack of consistency in how resilience is defined, who is involved (equity and inclusivity), when and where it occurs (across temporal and spatial scales), and how it impacts. While it is common to see the juxtaposition of concepts across disciplines, the application and appropriation of terms without a common understanding can highlight a lack of awareness among scholars and practitioners. Broad disciplinary usage can reinforce a sense of ambiguity and potentially dilutes the real‐world impact. The existing community resilience literature suggests community resilience risks becoming meaningless in the absence of a unifying understanding (Brand and Jax, 2007; Patel et al., 2017; Meerow and Newell, 2019).

### 1.4 Practice‐based versus academic approaches

A previous systematic review by the authors (Boston et al., 2024) identified contrasting applications of community resilience across academia and in practice‐based literature. An evaluation of 24 practice‐based guidelines, such as those developed by the United Nations Office for Disaster Risk Reduction and the Resilient Cities Network, found that practice‐based guidelines generally emphasise operational clarity, defining resilience through practical strategies, measurable indicators, and governance mechanisms. These frameworks often present resilience as a process of preparation, response, and recovery, with equity, inclusion, and community engagement identified as essential components. Many strategies adopt a top‐down or city‐scale orientation, but several also incorporate bottom‐up approaches, for applying resilience at smaller scale. The focus of practice‐based community resilience tends to be on implementation and outcomes that can be monitored by governments or institutions.

In contrast, academic literature on community resilience typically interrogates the conceptual foundations: debating resilience as equilibrium, adaptation, or transformation; examining whether it should be considered a process or an outcome; and unpacking the role of equity, governance, and social capital (Cutter et al., 2008; Norris et al., 2008; Matyas and Pelling, 2015; Boston et al., 2024). The academic framing highlights the pluralistic and multifaceted nature of the concept, varying from engineering notations of equilibrium (Bruneau et al., 2003), to ecological adaptation (Carpenter et al., 2001; Engle, 2011), to sociopolitical perspectives centred on equity and transformation (Folke et al., 2002; Nagenborg, 2019). In contrast, practice‐based frameworks distil these ideas into actionable guidance for policy and community implementation.

### 1.5 The case for transdisciplinary approaches

Recognising that no discipline alone can solve the complex challenges facing communities and valuing community within a human settlement context as multifaceted, encompassing citizens, policymakers, and governments, businesses, and charitable organisations, there is a need to better understand and address the ambiguity of the term community resilience to support and enable collective action. Yet, this paper does not suggest that community resilience aims to create a homogeneous community, or that the disciplinary perspectives of community resilience should become obsolete. Rather, in seeking a sense of understanding that transcends disciplines, we assert that the value of community resilience is in integrating individual perspectives. This is what Thorén (2014) describes as a ‘unifying concept’, Star and Griesemer (1989) define as a ‘boundary object’, and Olsson et al. (2015) depict as pluralistic problem feeding. A broad approach to understanding and practising resilience is necessary to encourage equity across participation, community capacity, and capital, particularly to avoid it becoming a colonial or neoliberalism term (Cretney, 2014).

Accounts of transdisciplinary problem‐solving in communities exist in the literature (Stokols, 2006; Polk, 2014; Cundill, Roux, and Parker, 2015; Foshag, 2020; Caramel, 2021), with Oyinlola et al. (2018) suggesting that transdisciplinary teams are better equipped to respond to complex challenges pragmatically. However, challenges exist in translating knowledge for realised and meaningful change (Polk, 2014; Shah, Paulson, and Couch, 2020). A better understanding of how transdisciplinarity is understood can potentially address this challenge and contribute to community resilience.

### 1.6 Aims and contribution of the study

This study aims to capture, collate, and discuss the perspectives of cross‐disciplinary practitioners to explore how the concept of community resilience is understood, defined, and applied. An improved understanding of transdisciplinary community resilience may support researchers, practitioners, and community leaders across disciplines in engaging more effectively in collective problem‐solving and action. In this vein, we also believe that there is a need to inform the design and development of innovative and contextually‐rich higher education educational offerings in community resilience to support this objective further (Brogden et al., 2022).

This study is part of a larger research project funded by the Royal Academy of Engineering's Frontiers' Programme,^1^ which seeks to (i) understand the complexities of community resilience as a boundary object and (ii) build an online open educational resource for use by educators looking to address issues of community resilience through teaching and learning. The research team is transdisciplinary and international, representing engineering, architecture, construction and surveying, urban planning, and design across six higher education institutions and architectural practice in five countries.

## 2 MATERIAL AND METHODS

This study adopted a quantitative approach, using an online survey to collect data that could help to better understand perspectives on resilience among international respondents from different disciplinary backgrounds. Online surveys are a low‐cost, accessible, time‐efficient, and flexible method that enables ‘access to data that range in focus from peoples’ views, experiences, or material practices, through representational or meaning‐making practices' (Braun et al., 2021, p. 2). The survey design drew on the community resilience capabilities described by Bruneau et al. (2003) and Norris et al. (2008), and the survey questions are available in the Appendix in the supplementary materials.^2^

The survey was created using Google Forms and was distributed via social media (Twitter (now ‘X'), Facebook, and LinkedIn) and by direct e‐mail to the professional networks of the research team members who were based in Australia, New Zealand, Nigeria, South Africa, and the United Kingdom, many of whom were already engaged in the field of resilience. Participation was voluntary, not restricted to any one country, and respondents remained anonymous. The distribution strategy was therefore opportunistic, with the response number reflecting both the size and level of engagement of the individual researchers' networks.

The survey addressed three thematic areas of interest:

1. About you: disciplinary context and involvement with community resilience.
2. About resilience: definitions of community resilience and relevance of resilience capacities.
3. About resilience education: future curriculum for community resilience education.

This paper reports the findings that emerged from the first two thematic areas (about you and about resilience) to focus on developing a common understanding of resilience across multiple disciplines and geographies. Insights from the third thematic area, about resilience education, are presented in a further paper and highlight practice‐based approaches for improving the teaching of resilience (Brogden et al., 2022).

## 3 SAMPLE GROUP

A total of 111 international participants completed the online survey during 2020, distributed most notably across Nigeria (49.1 per cent), the UK (15.7 per cent), South Africa (11.1 per cent), New Zealand (7.4 per cent), the United States of America (5.6 per cent), and Australia (2.8 per cent)—the remaining countries were Portugal, Sierra Leone, Tanzania, China, France, Gambia, Germany, Ghana, and Mozambique. The large number of respondents from Nigeria is a limitation of the study that is unintentional. Participants represented six professional or vocational contexts, with the most significant proportion engaged in an educational setting as ‘Educator/Academic’ (63.9 per cent) or ‘Researcher’ (15.7 per cent). The remainder comprised ‘Practitioner/Professional’ (15.7 per cent), ‘Government’ (2.8 per cent), ‘Non‐Profit’ (1.9 per cent), and ‘Volunteer’ (0.9 per cent).

Respondents were also asked to define their current field or discipline. The majority of participants self‐identified as working in fields related to the built environment, including, in particular, ‘Urban Planning’ (30.6), ‘Social Sciences’ (18.5 per cent), ‘Environmental Science’ (9.3 per cent), ‘Architecture’ (9.3 per cent), ‘Engineering’ (7.4 per cent), and ‘Construction/Surveying’ (6.5 per cent)—the other disciplinary contexts represented were ‘Design’, ‘Urban Design’, ‘Software Design’, ‘Heritage Practice’, ‘Communication’, ‘Community Development’, ‘Social Sciences’, ‘Science’, and ‘Education’.

## 4 STATISTICAL ANALYSIS

Three statistical analysis methods were adopted using SPSS (Statistical Package for the Social Sciences) v.27 software, one‐way analysis of variance (ANOVA), Pearson's correlation, and inter‐rater reliability.

First, one‐way ANOVA was undertaken to identify whether significant differences in responses occurred between different groups of participants. The test assumes that each sample is an independent random sample and that the distribution of the response variable follows a normal distribution (that is, variance is homogeneous) (Field, 2009). Comparisons were made between independent groups of participants to ensure random sampling, while the homogeneity of variances hypothesis was checked using Levene's test, with a confidence interval set at 95 per cent. Homogeneity of variance was met in most cases. For the very few instances where it was not, one‐way Welch's ANOVA results were used, as these are not sensitive to unequal variances (Field, 2009).

Second, an analysis using Pearson's correlation was recognised as a useful approach for determining the strength and direction of the association between two scales or ordinal variables. Pearson's correlation coefficient measures linear association and is a test commonly used for parametric analysis (where data follow a specific structure or distribution such as normality) (Field, 2009). Positive correlations indicate that if one variable increases, the other variable increases as well. Conversely, negative correlations indicate that if one variable increases, the other variable decreases.

Third, inter‐rater reliability was utilised to test consistency of agreement between raters. Krippendorff's alpha model was used (a two‐way mixed model with a 95 per cent confidence interval). This provided an intraclass correlation coefficient (ICC) of average measures, where inter‐rater reliability is rated as acceptable for values greater than 0.7, good for values greater than 0.8, and excellent for values greater than 0.9 (Field, 2009).

For both one‐way ANOVA and Pearson's correlations, the results were considered to be statistically significant for *p* < 0.05 and *p* < 0.01, where *p* is the significance value. Furthermore, effect sizes were computed using eta squared (*η* ^2^) for one‐way ANOVA results that were statistically significant. Such effect sizes can be categorised as small (0.2), medium (0.5), or large (0.8), using Cohen's scale (Cohen, 1988). It is important to note that small effect sizes indicate that differences observed are trivial, even if statistically significant. Therefore, it is critical to report effect sizes (*η* ^2^) in addition to significance values (*p*) for one‐way ANOVA results.

## 5 RESULTS

### 5.1 Defining community resilience

To explore how community resilience is understood and defined, respondents were asked to identify the first, second, and third words that came to mind when thinking about community resilience. These questions required open responses to avoid leading participants in their definitions.

A total of 91 answers were given for the ‘first‐word’ question, and Table 1 shows those words occurring at least twice. The high number of responses and low percentages shown in Table 1 indicate no established keywords associated with community resilience across the participant group. However, some words did appear regularly, including ‘Adaptation’, ‘Sustainability’, ‘Survival’, and ‘Strength’ (or variants of those), although percentages of occurrence were all below 10 per cent.

**TABLE 1:** First words coming to mind when thinking about community resilience.

First word	Frequency of occurrence	Total frequency of occurrence (number – %)
Adaptation / Adaptability	4 / 4	8 – 8.8%
Sustainability / Sustainable / Sustainable development / Sustainable resources	3 / 1 / 1 / 1	6 – 6.6%
Survival / Survival in the face of stress / Survive	3 / 1 / 1	5 – 5.5%
Strength / Strong	2 / 1	3 – 3.3%
Coping / Coping strategies	1 / 1	2 – 2.2%
Ability / Ability to carry on in the midst of problems or challenges	1 / 1	2 – 2.2%
Recovery / Recover	1 / 1	2 – 2.2%
Readiness to any shock / Shocks	1 / 1	2 – 2.2%
Support / Social support	1 / 1	2 – 2.2%
Security / Food security	1 / 1	2 – 2.2%
Resources / Sustainable resources	1 / 1	2 – 2.2%
Connection / Connectedness	1 / 1	2 – 2.2%
Trust	2	2 – 2.2%

**Source:** authors.

A total of 97 answers were given for the ‘second‐word’ question, and Table 2 shows words with at least two occurrences. Again, a high number of answers and low percentages indicate no established keywords. Furthermore, ‘Bouncing back / Rebound’ and ‘Ability to withstand / Ability to respond / Ability to maintain’ were found to be common first and second words.

**TABLE 2:** Second words coming to mind when thinking about community resilience.

Second word	Frequency of occurrence	Total frequency of occurrence (number – %)
Adaptation / Adaptive / Adaptability / Adaptiveness / Adaptive strategies	1 / 2 / 1 / 1 / 1	6 – 6.2%
Bouncing back / Bounce back / Fighting back / Rebound / Back to normal	1 / 1 / 1 / 1 / 1	5 – 5.2%
Ability to withstand / Ability to respond / Ability to maintain / Ability	1 / 1 / 1 / 1	4 – 4.1%
Sustainable / Sustainability	1 / 2	3 – 3.1%
Survival / Survival or Coping	2 / 1	3 – 3.1%
Social capital / Social cohesion	2 / 1	3 – 3.1%
Preparedness	3	3 – 3.1%
Support	3	3 – 3.1%
Disaster / Moving forward after a disaster	2 / 1	3 – 3.1%
Endurance	2	2 – 2.1%
Capacity / Capacity to act/mitigate	1 / 1	2 – 2.1%
Cohesion / Social cohesion	1 / 1	2 – 2.1%
Durable / Durable shelter	1 / 1	2 – 2.1%
Power / Empowering	1 / 1	2 – 2.1%
Development / Development of people and places	1 / 1	2 – 2.1%
Flexibility	2	2 – 2.1%
Withstand / Ability to withstand	1 / 1	2 – 2.1%
Collaboration / Collaborative	1 / 1	2 – 2.1%
Preparation	2	2 – 2.1%

**Source:** authors.

A total of 100 answers were given for the ‘third‐word’ question, and Table 3 shows those with at least two occurrences. In addition to the first and second words already mentioned, ‘Recovery’ and ‘Resourcefulness’ were also identified as third words coming to mind when thinking about community resilience.

**TABLE 3:** Third words coming to mind when thinking about community resilience.

Third word	Frequency of occurrence	Total frequency of occurrence (number – %)
Sustainability / Sustainability of people and places / Sustainable	2 / 1 / 2	5 – 5%
Adaptability / Adaptation	2 / 3	5 – 5%
Recovery / Ability to withstand or recover	2 / 1	3 – 3%
Survival / Survive	2 / 1	3 – 3%
Resourcefulness / Resources	2 / 1	3 – 3%
Regaining strength / Strength / Strong willed	1 / 1 / 1	3 – 3%
Strategies / Clear strategies	1 / 1	2 – 2%
Build back better / Rebuilding	1 / 1	2 – 2%
Community support / Growth of community	1 / 1	2 – 2%
Together / Togetherness	1 / 1	2 – 2%
Creative / Creating	1 / 1	2 – 2%
Common goals / Commons	1 / 1	2 – 2%
People	2	2 – 2%
Ability to withstand and recover / Withstand	1 / 1	2 – 2%
Regain / Regaining strength	1 / 1	2 – 2%

**Source:** authors.

When combining the results of the first, second, and third words, it was found that the most common were ‘Adaptation’, ‘Sustainability’, ‘Bouncing back’, and ‘Survival’ (or variants thereof). Nevertheless, the percentages of these words were low (well below 10 per cent).

### 5.2 Characteristics of community resilience

The next section of the survey asked respondents to comment upon a set of four characteristics for increasing community resilience—first identified by Bruneau et al. (2003) and extended by Norris et al. (2008). The characteristics are:

1. Robustness: the ability to withstand stress without suffering degradation.
2. Redundancy: the extent to which elements are substitutable in the event of disruption of degradation.
3. Rapidity: the capacity to achieve goals promptly to contain losses and avoid disruption.
4. Resourcefulness: the capacity to identify problems and mobilise resources when conditions threaten the system.

Respondents were asked to rate the importance of each characteristic using a scale of one to five (where one equals not important and five equals very important).

Inter‐rater reliability analysis yielded an excellent ICC of 0.940 for the answers provided across those questions. The results suggest that respondents perceived all of the requirements mentioned as either ‘important’ (rating of four) or ‘very important’ (rating of five), as shown in Table 4.

**TABLE 4:** Requirements ratings for community resilience (‘important’ and ‘very important’ percentage results).

Requirement	Important	Very important	Total
Robustness	38.9%	50.0%	**88.9%**
Redundancy	38.0%	37.0%	**75.0%**
Rapidity	44.4%	40.7%	**85.1%**
Resourcefulness	20.4%	74.1%	**94.5%**

**Source:** authors.

The most important requirement was found to be ‘Resourcefulness’, followed by ‘Robustness’. Three‐quarters of respondents found ‘Resourcefulness’ to be very important, well above ‘Robustness’, which was rated as very important by one‐half of the respondents.

One‐way ANOVA results show that the professional/vocational context significantly affected the ‘Robustness’ question (*F*(3,104) = 3.630, *p* = 0.015). In particular, Researchers rated ‘Robustness’ lower than any other group (3.9/5); in comparison, Educators/Academics rated it as 4.5/5, Practitioners/Professionals as 4.2/5, and all remaining participants as 4.3/5. One should note that a rating of four corresponds effectively to ‘important’, while five corresponds to ‘very important’, suggesting that such differences, although statistically significant, have little practical implication.

One‐way ANOVA results show that the country where participants were based significantly affected the ‘Rapidity’ question (*F*(6,101) = 5.441, *p* = 0.000). The results obtained were: Nigeria, 4.5/5; UK, 4.1/5; South Africa, 3.8/5; New Zealand, 4.0/5; US, 4.7/5; Australia, 2.7/5; and any other country, 4.3/5. They show that Australia's rating was significantly lower than the five other countries. Still, it is important to point out that only three participants from Australia answered the survey, so the difference observed with the Australia rating is not statistically relevant. All other ratings were close to four or five, suggesting that the differences observed have little practical implication.

The correlation analysis presented in Table 5 shows that statistically significant correlations (*p* < 0.05) exist between most of the requirements, but the strength of correlations (that is, the *r* values) are low. For instance, the strongest correlation was found between ‘Redundancy’ and ‘Rapidity’.

**TABLE 5:** Pearson correlation coefficients (*r*) and significance of correlations between the requirements for community resilience.

	Robustness	Redundancy	Rapidity	Resourcefulness
**Robustness**	1	0.19*	0.242*	0.196*
**Redundancy**	0.19*	1	0.313**	0.221*
**Rapidity**	0.242*	0.313**	1	0.029
**Resourcefulness**	0.196*	0.221*	0.029	1

**Notes:**

* Correlation is significant at the 0.05 level.

** correlation is significant at the 0.01 level.

**Source:** authors.

### 5.3 Networked resources for community resilience

Next, participants were introduced to networked resources for resilience (Norris et al., 2008) and asked to rank each in order of importance:

- ‘Economic Development’: for example, to be equitable, stable livelihoods, and to be wealthy.
- ‘Social Capital’: for example, individuals are connected and support each other.
- ‘Information and Communication’: for example, these systems operate to tell everyone what they need to know.
- ‘Community Competence’: for example, collective action and decision‐making.

These networked capacities were based on a review of existing literature. The first two resources, ‘Economic Development’ and ‘Social Capital’, are structural characteristics of a community and can be measured using publicly available data (Sherrieb et al., 2010). The second two capacities, ‘Information and Communication’ and ‘Community Competence’, are based on community or government processes and are harder to quantify.

Inter‐rater reliability analysis provided a good ICC of 0.850 for the rating of networked resources. Overall, the ranking of networked resources in order of importance was found to be:

1. ‘Social Capital’.
2. ‘Information and Communication’.
3. ‘Community Competence’.
4. ‘Economic Development’.

**FIGURE 1:**
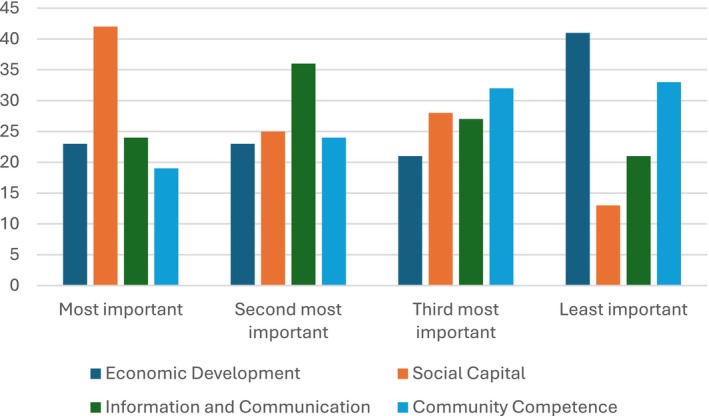
Rating of networked resources in order of importance. **Note:** the bars equal the number of responses. **Source:** authors.

It should be noted, however, that no strong consensus was found for those ratings, as the percentage of responses for each was always less than 50 per cent: 39 per cent chose ‘Social Capital’ as most important; 33 per cent chose ‘Information and Communication’ as second most important; 30 per cent chose ‘Community Competence’ as third most important; and 38 per cent chose ‘Economic Development’ as least important (see Figure 1).

One‐way ANOVA results show that not being involved in resilience education or somewhat involved significantly affected the answers provided for the most important networked resources (*F*(1,106) = 6.426, *p* = 0.013). The ranking for those not involved was (‘most important’ question): (i) ‘Economic Development’, 29.8 per cent; (ii) ‘Social Capital’, 27.7 per cent; and (iii) ‘Information and Communication’ and ‘Community Competence’, 21.3 per cent each. The ranking for those involved was (‘most important’ question): (i) ‘Social Capital’, 47.5 per cent; (ii) ‘Information and Communication’, 23.0 per cent; (iii) ‘Community Competence’ and ‘Economic Development’, 14.8 per cent each. These results indicate that those not involved in resilience education did not have a clear view of which are the most important networked resources, as percentages are all within about nine per cent of one another, whereas those involved had a more clearly defined view, with the most cited resource being about 33 per cent higher than the least cited.

### 5.4 Collective resilience

**FIGURE 2:**
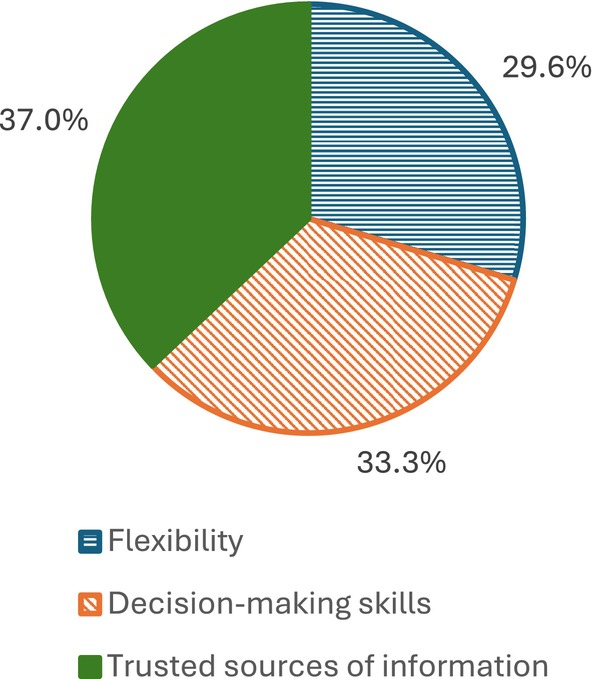
Most important factor for building collective resilience. **Source:** authors.

Norris et al. (2008) argue that building collective resilience requires three factors that function in the face of unknowns: ‘Flexibility’; ‘Decision‐making skills’; and ‘Trusted sources of information’. Respondents were asked to identify what they considered to be the most important factor (see Figure 2).

Figure 2 shows a fairly even split of responses for the three factors mentioned, with a slightly higher percentage accorded to ‘Trusted sources of information’ in terms of importance.

### 5.5 Adaptive capacities

Respondents were also asked to consider five recommendations for increasing community resilience (Norris et al., 2008) and to indicate the extent to which they agreed or disagreed with the following statements:

- First, to increase their resilience to disaster, communities must develop economic resources, reduce risk and resource inequities, and attend to their areas of greatest social vulnerability.
- Second, to access social capital, one of the primary resources of any community, local people must be engaged meaningfully in every step of the mitigation process.
- Third, pre‐existing organisational networks and relationships are the key to rapidly mobilising emergency and ongoing support services for disaster survivors.
- Fourth, interventions are needed that boost and protect naturally occurring social supports in the aftermath of disasters.
- Fifth, communities must plan, but they must also plan for not having a plan; this means that communities must exercise flexibility and focus on building effective and trusted information and communication resources that function in the face of unknowns.

**FIGURE 3:**
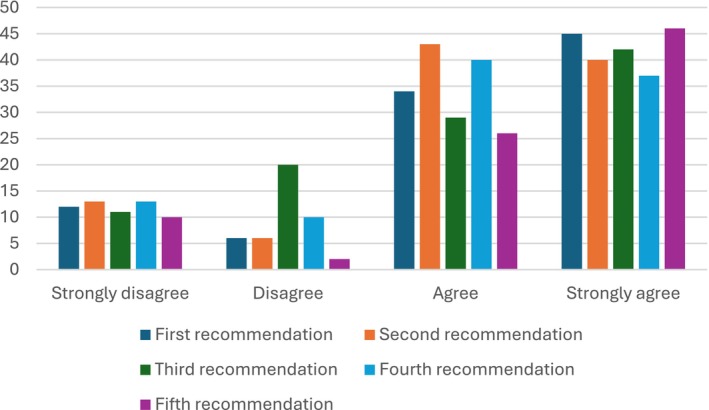
Agreement with the recommendations of Norris et al. (2008). **Note:** the bars equal the number of responses. **Source:** authors.

Overall, very good agreement was found with the statements of Norris et al. (2008), with a clear majority of ‘Agree’ or ‘Strongly agree’ for all the recommendations given (‘First recommendation’ = 81 per cent; ‘Second recommendation’ = 81 per cent; ‘Third recommendation’ = 73 per cent; ‘Fourth recommendation’ = 77 per cent; and ‘Fifth recommendation’ = 86 per cent). Percentages were also comparable (73–86 per cent range), suggesting that all of the recommendations given were found to be roughly equally important (see Figure 3).

## 6 DISCUSSION

### 6.1 Disciplinary definitions and ambiguity

**FIGURE 4:**
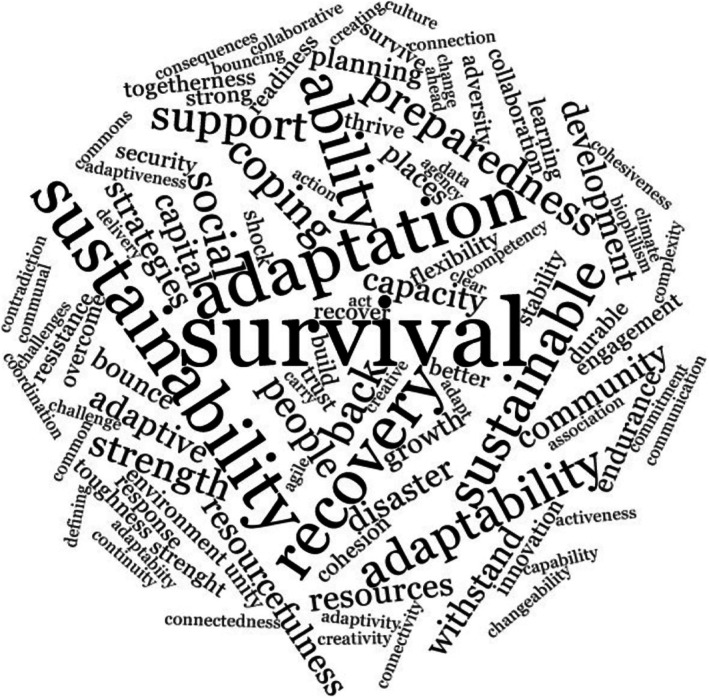
Word cloud of emerging resilience keywords. **Source:** authors.

The findings suggest a lack of common language around community resilience, with no established keywords within or across disciplinary contexts. When asked to identify first, second, and third words that came to mind when thinking about community resilience, the respondents provided 288 words with a low frequency of occurrence. Four words that appeared to show some common understanding across participants, albeit with a low incidence, were ‘adaptation’, ‘sustainability’, ‘bouncing back’, and ‘survival’ (see Figure 4).

These terms, which align with recent scholarly work by Bhandari and Alonge (2020) and Nguyen and Akerkar (2020), suggest that core definitions of community resilience are absorption capacity, restorative capacity, robustness, fault tolerance, flexibility, survivability, and agility. While they represent common thinking among respondents, they can still be considered ambiguous; application extends across disciplines and beyond the field of community resilience, and indeed resilience in its broadest sense. This finding indicates that although disciplinary perspectives can offer a rich and nuanced understanding of community resilience, translation into common shared understanding across disciplines is limited. Without a common understanding, it can be challenging to translate resilience into action or tangible outcomes for a community.

Despite the ambiguity, respondents reached a stronger sense of common understanding when given a description that was contextualised in practice. They agreed on the adaptive capacities required to increase community resilience: the five recommendations offered by Norris et al. (2008). This suggests that providing respondents with the language and meaning to articulate community resilience can support them both in understanding and agreeing on a collective approach. The supply of resources that extend knowledge and understanding of the what and how of community resilience, grounded contextually in practice, might therefore support the emergence of a transdisciplinary definition of community resilience.

Our results revealed little consensus among practitioners on how resilience should be defined or operationalised, whether framed in terms of system characteristics (robustness, redundancy, rapidity, and resourcefulness—the ‘4Rs’), networked resources (economic development, social capital, information and communication, and community competency), collective resilience, or adaptive capacity. This lack of agreement reflects broader tensions and political reluctance around systemic thinking and challenges between the operational and conceptual views found in resilience literature. Connolly (2018) contends that institutional rules, norms, and incentives create entrenched definitions of what is desirable, limiting the scope for systemic change and helping to explain political reluctance to adopt systemic framing in resilience. Similarly, Helfgott (2018) highlights that while resilience serves as a boundary object with the potential to link disciplines, its very flexibility generates tensions between operational uses focused on recovery and conceptual perspectives centred on adaptation and transformation. Mukkarram Munawwar Ali and Jones (2013) further show how political priorities and lived experiences blur the line between proactive and reactive adaptation, reinforcing context‐specific interpretations of resilience. Wilson (2014) adds that path dependencies (social, economic, cultural, and psychological) constrain the possibilities for systemic change, making consensus difficult to achieve. Together, these insights suggest that the absence of a shared definition in our findings is not unique to our study, but reflects deeper institutional, disciplinary, and political dynamics that continue to shape how resilience is understood and applied.

While resilience continues to serve as a boundary object that unites diverse disciplines yet resists a singular definition (Boston et al. 2024), it is nonetheless possible to situate specific resilience dimensions within established scholarly literature. To this end, we map the four focus areas of our study (characteristics of resilience, networked resources, collective resilience, and adaptive capacity) against prominent frameworks, system perspectives, and governance approaches (see Table 6).

**TABLE 6:** Mapping of the study focus areas on to broader resilience scholarship*.

Study section	Frameworks	Complexity/systems thinking	Adaptive governance
**Defining resilience**	No single definition; boundary object across disciplines (Cutter et al., 2008; Patel et al., 2017; Helfgott, 2018; Boston et al., 2024).	Frames resilience as a contested yet flexible concept.	Provides integrative policy lens.
**Characteristics of resilience (the Rs)**	Engineering resilience frameworks (Bruneau et al., 2003; Almutairi et al., 2020; Chee Yin, Kassem, and Mohamed Nazri, 2022).	Stabilising/destabilising dynamics (Therrien, Normandin, and Denis, 2017); ecological resilience (Holling, 1973).	Not applicable; less of a governance focus.
**Networked resilience**	Community‐centred frameworks (Norris et al., 2008; Patel et al., 2017; Vaneeckhaute et al., 2017).	Resilience emerges from networks and institutions (Cox, Jr., 2012).	Cross‐scale linkages to institutions.
**Collective resilience**	Community resilience 2.0 (Vaneeckhaute et al., 2017).	Group‐level decision‐making (Cox, Jr., 2012).	Trust, shared learning, and collective problem solving (Choudhury, Haque, and Doberstein, 2021).
**Adaptive capacity**	Multidimensional resilience frameworks (Ostadtaghizadeh et al., 2015).	Feedback loops and adaptive cycles (Therrien, Normandin, and Denis, 2017).	Adaptive governance through social learning and institutional flexibility (Folke et al., 2002; Hall, 2014; Choudhury, Haque, and Doberstein, 2021).

**Note:**

* The table illustrates how each area identified in this study corresponds to established resilience frameworks, complexity and systems thinking, and adaptive governance perspectives.

**Source:** authors.

The first focus area of our study, characteristics of resilience, aligns with engineering‐oriented frameworks that emphasise quantifiable system properties (Almutairi, Mourshed, and Ameen, 2020; Chee Yin, Kassem, and Mohamed Nazri, 2022). Yet, complexity perspectives highlight that the 4Rs of resilience interact in a dynamic way as stabilising and destabilising forces (Therrien, Normandin, and Denis, 2017), echoing Holling's (1973) foundational insight that resilience is not only about returning to equilibrium but also about navigating cycles of disruption and renewal. This resonates with our finding of resourcefulness being the most favourable characteristic as a means to achieve change and improve outcomes.

Second, networked resources align well with community‐centred frameworks that stress the importance of social relationships, knowledge sharing, and collective agency (Norris et al., 2008; Patel et al., 2017; Vaneeckhaute et al., 2017). This corresponds with our survey finding that ranked social capital and information and communication as the most important attributes of networked resources. System thinking reinforces this perspective by showing how resilience emerges from the configuration of networks and institutions rather than individual components.

Third, collective resilience encompasses flexibility, decision‐making skills, and reliance on trusted sources of information. This extends beyond individual or organisational resilience to the capacity of groups to deliberate, coordinate, and act under uncertainty. Our study found equally favourable responses to the three areas of collective resilience. Frameworks such as ‘Resilience 2.0’ highlight the role of communal agency and social memory in shaping resilience pathways (Vaneeckhaute et al., 2017), while adaptive governance perspectives emphasise the importance of trust and shared decision‐making in collaborative problem solving (Choudhury, Haque, and Doberstein, 2021). System thinking also recognises collective decision‐making as a driver of emergent resilience, although Cox, Jr. (2012) cautions that aggregating individual preferences does not always yield a desirable outcome.

Lastly, respondents broadly endorsed the five adaptive capacity statements of Norris et al. (2008), reinforcing the importance of learning, flexibility, and proactive adjustment. Complexity theory situates adaptive capacity as the community's ability to navigate between stabilising and destabilising forces, enabling continuous adjustment to shifting conditions (Therrien, Normandin, and Denis, 2017). Systems approaches accentuate temporal dimensions where adaptation unfolds dynamically as communities absorb lessons from past disruptions and anticipate future risks (Nguyen and Akerkar, 2020). Adaptive governance extends this to institutions, framing adaptation as an iterative process of multilevel collaboration, reflexivity, and social learning (Choudhury, Haque, and Doberstein, 2021). The alignment between our findings and Norris et al.'s (2008) framework underscores, therefore, adaptive capacity as a bridging dimension that connects individual, social, and institutional levels of resilience.

While the characteristics of resilience (the Rs) align most closely with engineering‐based frameworks, networked resources and collective resilience resonate with community‐centred approaches that underline social networks, agency, and trust. Adaptive capacity connects directly to adaptive governance and complexity‐informed perspectives that stress feedback learning and institutional flexibility.

This study addresses the divergence between abstract conceptualisations of resilience (as captured in frameworks, systems thinking, and adaptive governance theory) and the operational priorities identified by communities. While frameworks highlight the importance of multidimensional and dynamic interactions, our results show how respondents prioritise resourcefulness, social capital, and trusted information as practical anchors of resilience. This suggests a way to bridge the gap between theoretical models and community‐grounded, operational understandings of resilience.

### 6.2 The role of resourcefulness

Among the community resilience characteristics discussed (the 4Rs), resourcefulness was most favoured, with 94.5 per cent of respondents identifying this requirement as either important or very important. Described as ‘the capacity to identify problems and mobilize resources when conditions threaten the system’ (Norris et al., 2008, p. 134), resourcefulness can be understood as closely aligned with the most common descriptor identified: adaptation.

Resourcefulness and adaptation are similar in that they represent forms of agency; however, these forces require pinpointing and practising an approach underpinned by capacity building and education. This contrasts with other characteristics such as robustness and redundancy, which are more closely aligned with the physical characteristics of the community before disruption, and the capacity to resist it without declines in functionality.

In the built environment, current design codes and standards focus primarily on life safety or the resistance and robustness of a system (such as a building or infrastructure network). Structures meeting these standards are robust enough not to collapse but they may lose some or all functionality—for example, a building is resilient if it is not damaged and can continue to be used after the disruption (Grigorian et al., 2019). The survey results indicate, though, the importance of resourcefulness as a measure of a community's ability to adapt and suggest that this might be a space for further transdisciplinary consideration.

Experience of disasters triggered by natural hazards, climate change, and possibly COVID‐19 (coronavirus disease 2019) have demonstrated that even with a modern and robust built environment, the recovery process can be long and difficult, and communities may be forced to adapt and live with their altered surroundings for many years. While it should remain important for our built environment to be robust, the preservation does little to help our community if it lacks the resourcefulness to cope with and adapt to other changes presented by disruptions. Thus, it may be prudent to rethink the resilient design of communities to consider how people and organisations can adapt and change in the face of disruption.

### 6.3 Towards collective resilience

Agreement across the three components of collective resilience, flexibility, decision‐making skills, and trusted sources of information, correlate with that of Norris et al. (2008), suggesting that these factors can be considered as transcending disciplines. To this end, efforts that attempt to encourage transdisciplinary understanding of community resilience should be framed around terms that are relevant and understood in a disciplinary sense.

Additionally, the acknowledgement of social capital as the most important networked resource suggests that a greater focus on the philosophy underpinning community design could help to reinforce notions of community resilience. This aligns with Imperiale and Vanclay (2021), who identify community resilience as cognitive and interactional social processes.

Recognising, valuing, and capitalising on social capital could strengthen communities and support collective action, foregrounding a transdisciplinary response to challenges faced. Yet, it was clear in the findings that those involved in resilience had a much stronger appreciation of the potential of networked resources. This highlights the possibility for transdisciplinary education in community resilience, offering practice‐based examples to share knowledge, raise awareness, and generate community impact.

## 7 CONCLUSION

This study aimed to offer a better understanding of international and cross‐disciplinary perspectives of community resilience. The findings suggest that there is little distinction between how community resilience is described across disciplines or geographic locations, with all participants acknowledging that the term is difficult to articulate. In this way, how we understand community resilience can be considered a global and transdisciplinary issue and may create challenges in how policy and planning decisions are made and implemented.

This research further reinforces the known ambiguity of the term community resilience, indicating that there was little agreement on keywords and terms among respondents. However, when presented with a framework such as adaptive capacities, respondents were able to identify with and describe community resilience using the language presented. This suggests that sharing examples of community resilience from practice, together with disciplinary contextual framings, could inform and support a stronger transdisciplinary understanding of community resilience, particularly among people who are uncertain of their individual or disciplinary interpretation.

Importantly, the analysis revealed that a transdisciplinary understanding of community resilience is underpinned by human capacity and agency—demonstrated here through the demand for resourcefulness, the role of social capital, and the components of collective resilience. With this in mind, valuing the agency of individual capabilities and demonstrating the characteristic of resourcefulness can inform transdisciplinary community resilience and support the generation of collective action.

It is recognised in the literature that practice‐based guidelines lack clarity, which is hindered by an inability to frame resilience effectively. Practical strategies are often top‐down and are reduced to factors that are able to be distilled into ‘measurable outcomes and indicators’. Conversely, academic‐derived information attempts to focus conceptualised debates on what constitutes resilience and much dialogue centres on whether it is a ‘process’ or an ‘outcome’. This work aimed to bridge the academic and practical or actionable resilience realms, acknowledging the need for common language to attain better outcomes. Indeed, ambiguity hampers collective action.

Future policy development and planning should recognise the benefits of transdisciplinary teams that have been shown to respond pragmatically to complex challenges. This work attempted to attain perspectives on resilience from geographically and disciplinary diverse backgrounds. Political reluctance to embrace ‘systems thinking’ that better reflects the complexity of community resilience often fails to integrate conceptual views into operational delivery. The importance of ‘adaptive capacity’ that dimensionally connects individuals across institutional levels should be embedded in policy. Indeed, as mentioned, this study addressed the divergence between abstract conceptualisations of resilience (as captured in frameworks, systems thinking, and adaptive governance theory) and the operational priorities identified by communities. While frameworks highlight the importance of multidimensional and dynamic interactions, our results show how respondents prioritise resourcefulness, social capital, and trusted information as practical anchors of resilience. This points to a way to bridge the gap between theoretical models and community‐grounded, operational understandings of resilience.

## CONFLICT OF INTEREST STATEMENT

None.

## ETHICS STATEMENT

Ethical approval for this study was granted by the School of Energy, Geoscience, Infrastructure and Society of Heriot‐Watt University.

## FUNDING STATEMENT

This work was supported by the Royal Academy of Engineering's ‘Frontiers’ programme (number: FoDT3\192021).

## Supporting information


**Data S1** Supporting Information

## Data Availability

The data that support the findings of this study are available from the corresponding author upon reasonable request.

## Endnotes

^1^
For more information, see https://raeng.org.uk/programmes-and-prizes/programmes/international-programmes/frontiers/ (last accessed on 1 December 2025).

^2^
All supplementary materials can be found online in the supporting information section at the end of the article.
