# Supplementary material for: Exploring disciplinary perspectives on community resilience
Source: Disasters. 2025 Dec 22;50(1):e70036. doi: 10.1111/disa.70036 (PMC12720063; doi:10.1111/disa.70036)
Supplement: Supplementary file 1 — Data S1 Supporting Information [file DISA-50-e70036-s001.docx]

**APPENDIX: Survey Questions**

**Section 1 – Education for a Resilient Future**

This survey aims to capture the views of stakeholders on what they would define ‘of value’ in the development and design of a prospective, innovative, contextually rich, university educational offering on the subject community resilience. It forms part of a project supported by the Royal Academy of Engineering under the Frontiers of Development Scheme. The project aims to explore and develop transdisciplinary frameworks for resilient design education targeting university students across a range of study areas.

The results of the questionnaire will help inform the development of the innovative educational offering and will also be used to frame a subsequent workshop on the topic. The questionnaire has been designed to ensure that no individual can be traced back to answers ensuring anonymity. No names will be used in any usage of the data which will be presented entirely anonymously. Participation in this study is completely voluntary. If you decide not to participate there will not be any negative consequences, and if you decide to participate, you may stop participating at any time. Ethical approval for this research was obtained from Heriot-Watt University EGIS Ethics Committee.

We are keen to hear your thoughts, opinions, and ideas on the potential role of education to support resilient futures. This short survey should take no more than a few minutes to complete.

**Section 2 – About you?**

This section asks about your context and involvement with community resilience.

What is your current professional/vocational context?

What is your current field/discipline?

What is your involvement in resilience education?

In which country are you currently based?

**Section 3 – About resilience**

This section asks about your response to resilience and the most important elements required for creating a common understanding of resilience.

We refer to the highly cited paper: Norris, F.H., Stevens, S.P., Pfefferbaum, B., Wyche, K.F., and Pfefferbaum, R.L., 2008. Community resilience as a metaphor, theory, set of capacities, and strategy for disaster readiness. American journal of community psychology, 41(1-2), pp.127-150.

*1. DEFINING COMMUNITY RESILIENCE*

List three words that best describe community resilience for you.

What is the first word that comes to mind when you think of community resilience?

What is the second word that comes to mind when you think of community resilience?

What is the third word that comes to mind when you think of community resilience?

*2. REQUIREMENTS FOR INCREASED COMMUNITY RESILIENCE*

Norris et al. (2008) note ‘Robustness; Redundancy; Rapidity and Resourcefulness’ as key characteristics required to increase community resilience. On a scale of 1 (not important) to 5 (very important) highlight the degree to which you feel these characteristics are important.

Robustness is ‘the ability to withstand stress without suffering degradation’ – To what degree is this important for community resilience?

Redundancy is ‘the extent to which elements are substitutable in the event of disruption of degradation’ – so being able to replace things easily – To what degree is this important for community resilience?

Rapidity is ‘the capacity to achieve goals in a timely manner to contain losses and avoid disruption’ – To what degree is this important for community resilience?

Resourcefulness is ‘the capacity to identify problems and mobilize resources when conditions threaten the system’ – To what degree is this important for community resilience?

*3. NETWORKED RESOURCES*

Norris et al. (2008) comment on the need to have networked resources for resilience, and that there are principally four of these:

ECONOMIC DEVELOPMENT

e.g. to be equitable, stable livelihoods and to be wealthy.

SOCIAL CAPITAL

e.g. individuals are connected and support each other.

INFORMATION AND COMMUNICATION

e.g. these systems operate to tell everyone what they need to know.

COMMUNITY COMPETENCE

e.g. collective action and decision-making.

List the networked resources in order of importance (from most important to least important).

*4. COLLECTIVE RESILIENCE*

Norris et al. (2008) argues that building collective resilience requires flexibility, decision-making skills, and trusted sources of information that function in the face of unknowns.

Select the one factor that you consider the most important for building collective resilience.

*5. ADAPTIVE CAPACITIES TO ENHANCE COMMUNITY RESILIENCE*

Norris et al. (2008) note five key recommendations to increase community resilience.

FIRST, to increase their resilience to disaster, communities must develop economic resources, reduce risk and resource inequities, and attend to their areas of greatest social vulnerability.

SECOND, to access social capital, one of the primary resources of any community, local people must be engaged meaningfully in every step of the mitigation process.

THIRD, pre-existing organizational networks and relationships are the key to rapidly mobilizing emergency and ongoing support services for disaster survivors.

FOURTH, interventions are needed that boost and protect naturally-occurring social supports in the aftermath of disasters.

FIFTH, communities must plan but they must also plan for not having a plan; this means that communities must exercise flexibility and focus on building effective and trusted information and communication resources that function in the face of unknowns.

Indicate to what extent you agree or disagree with the statements above (Strongly disagree / Disagree / Agree / Strongly agree).
